# Retroperitoneal lipoma, a rare cause of pelvic mass in women

**DOI:** 10.22088/cjim.12.0.495

**Published:** 2021

**Authors:** Maliheh Arab, Somayyeh Noei Teymoordash, Maryam Talayeh, Abdolali Ebrahimi, Niloofar nakhlian, Narges Khatoon Tabatabaei Shoja, Masoomeh Raoufi

**Affiliations:** 1 Department of Gynecology Oncology, Imam Hossein Medical Center, Shahid Beheshti University of Medical Sciences, Tehran, Iran; 2 2.Department of Pathology, Imam Hossein Medical Center, Shahid Beheshti University of Medical Sciences, Tehran, Iran; 3 3.Department of Obstetrics and Gynecology, Imam Hossein Medical Center, Shahid Beheshti University of Medical Sciences, Tehran, Iran; 4 4.Department of Obstetrics and Gynecology, Shahid Sadoughi University of Medical Sciences, Yazd, Iran; 5 5.Department of Radiology, Imam Hossein Hospital, Shahid Beheshti University of Medical Sciences, Tehran, Iran

**Keywords:** Retroperitoneal Lipoma, Pelvic Mass, Fatty Tumor

## Abstract

**Background:**

Lipoma is a benign mesenchymal tumor of soft tissue that occurs in almost all parts of the body where fat normally exists. Retroperitoneal lipomas are very infrequent condition with about 20 cases represented in the literature since 1980. They usually present as an abdominal mass or with pressure symptoms to adjacent organs.

**Case Presentation:**

A 66-year-old, post-menopausal woman referred to Imam Hossein Medical Center due to abdominal pain. Abdominopelvic magnetic resonance imaging (MRI) revealed a large mass containing fat component without enhancement on the right side of the pelvis. Tumor markers were within normal ranges. The patient underwent laparotomy and a 12 cm retroperitoneal mass which was located on the iliopsoas muscle with extension into the inguinal canal was resected with pathology report of lipoma. There has been no recurrence after one year of follow-up since surgery.

**Conclusion:**

In the differential diagnosis of retroperitoneal pelvic mass at all ages, lipoma should be considered as a rare cause.

The wide spectrum of retroperitoneal neoplastic and non-neoplastic soft tissue masses, reflects the complex anatomy of this region. Of the retroperitoneal tumors, 80% are malignant with liposarcoma representing the most common histological type (45%). However, retroperitoneal lipoma is a rare disease with only few cases of this condition reported in literature. Lipomas are encapsulated benign tumors of adipose tissue which can manifest at any fat containing body part (1). Lipomas are reported in all age groups, and are typically described as asymptomatic, soft and mobile masses. Despite being slow-growing tumors, these may grow up to giant sizes. Even though their preoperative diagnosis is benign, the definite treatment is total mass excision with capsule (2).We describe a rare case of retroperitoneal lipoma in a post-menopausal woman who was referred to us with abdominal pain and pelvic mass.

## Case presentation

A 66-year-old multiparous woman, with a history of nine deliveries and post-menopausal for the last ten years with a pelvic mass was referred to Imam Hossein Hospital as a tertiary medical center in Tehran, Iran. The patient’s clinical symptoms including intermittent abdominal pain in the hypogastric region began about 3 weeks ago. Other associated symptoms included urinary frequency and urgency as well as stress incontinence for past two years. The patient underwent total hysterectomy and bilateral salpingo-oophorectomy about 8 years ago due to uterine prolapse. Her past history also included hypertension, hyperlipidemia, and well-controlled diabetes.

**Figure 1:**
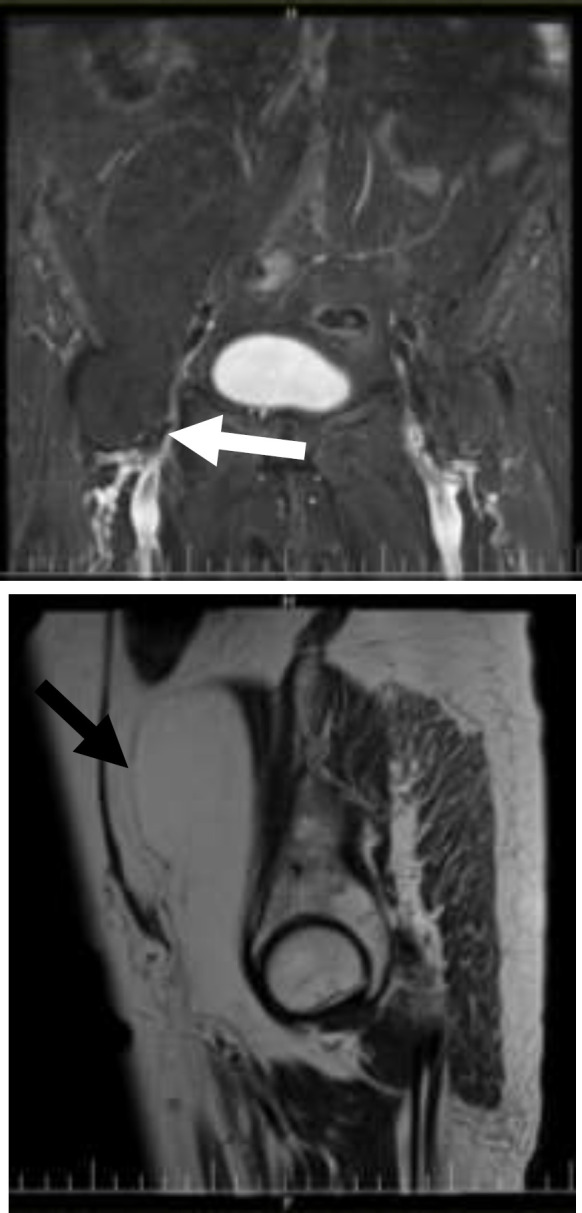
T2 coronal fat suppression (1) and T2 sagittal (1) images show dumbbell shape fat containing lesion extending from right retroperitoneal area to inguinal canal (white arrow) and suppressed fat in T2 coronal fat suppression image

In bimanual examination, left adnexal fullness was detected. On abdominal examination, we were unable to palpation of the mass due to the patient's obesity. Trans-abdominal ultrasonography showed an 84×103×112 mm heterogeneous mass with lobulated border on the right side of the pelvis extending to the right lower abdominal quadrant, with no vascular flow on color Doppler. Abdominopelvic magnetic resonance imaging (MRI) with and without contrast revealed a 150×77 mm large non-enhancing fat containing mass, suggestive of lipoma, located on the right side of pelvis extending along the anterior aspect of iliopsoas muscle (figure 1). Cancer antigen 125 (CA 125), carcinoembryonic antigen (CEA), human epididymal protein 4 (HE4), alpha-fetoprotein (AFP) were in the normal range, as well complete blood count (CBC).

**Figure2:**
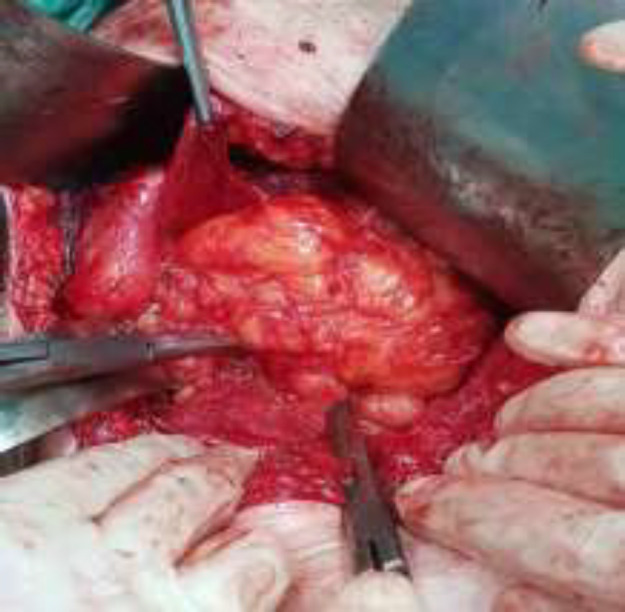
Large encapsulated retroperitoneal lipoma in right pelvic side extending towards inguinal canal

**Figure 3:**
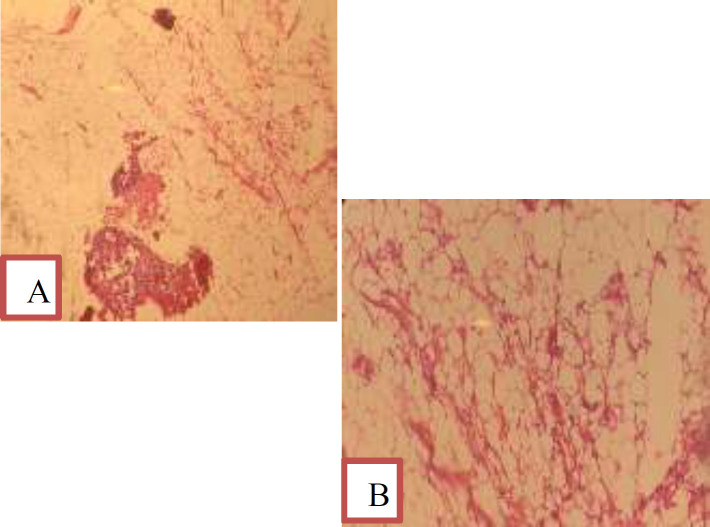
A) Lipoma include mature adipocyte and specific capillary network. Size of mature fat cells differ insignificant from each other. Thin fibrous septa separating the lobules are observed (Low magnification). B: H&E stain with high Magnification

The patient underwent laparotomy with midline incision. In the abdomen, a 12 cm long retroperitoneal mass was observed on the right side of the pelvis. The mass was located on the iliopsoas muscle, and extended to the inside of the inguinal canal. The mass had a lipoid appearance and consistency and was completely resected without rupture of capsule (figure 2). Lipoma was reported in the intraoperative frozen examination of the mass. Two days later, the patient was discharged without complications. Lipoma was confirmed in the final pathology report which indicated mature adipocyte, specific capillary network and thin fibrous septa separating the lobules (figure 3). During the follow-up, the patient had no problems after one year.

## Discussion

Lipoma is a benign encapsulated tumor of adipose tissue, mostly manifested in fifth and sixth decades of life and has no gender predominance (3, 4). The pathogenesis of lipoma has not yet been completely understood and both sporadic and inherited cases are observed. It is believed that the mesenchymal primordial fat tissue cells, and not the adult fat cells, are the cells of origin developing into lipoma (3). With regard the histopathological characteristics, lipomas are divided into subtypes, namely: conventional lipoma, angiolipoma, fibrolipoma, fusiform cell lipoma, myelolipoma and pleomorphic lipoma (1). Lipomas can appear in any fat containing body part including the trunk, limbs, mediastinum, and pelvis. While conditions like hypercholesterolemia, obesity, diabetes mellitus and trauma are considered the risk factors for development of the subcutaneous lipomas, data about such association is lacking for the retroperitoneal lipomas (5-7). 

Familial tendency is observed in some lipoma cases (8). Retroperitoneal lipoma might arise from the kidney, adrenal gland or retroperitoneal soft tissue. Deep lipomas, including those originating in the retroperitoneal space, are very infrequent with only fewer than 20 such cases published in the literature in the last 40 years (6). Pelvic retroperitoneal lipomas should be distinguished from pelvic lipomatosis; the latter being the fat proliferation in the perivesical and perirectal spaces of the pelvis (9). A lipoma is considered giant, when its diameter exceeds 10 cm. A pelvic lipoma may extend into the inguinal or perineal regions through the sciatic foramen, obturator foramen or pelvic floor (4).

These soft and mobile masses are typically asymptomatic and grow slowly; but over time, the pelvic lipomas can reach considerable sizes and become symptomatic (2). A diverse set of symptoms can result including: pelvic pain, dysuria, polyuria, hematuria (less frequently), urgency, urinary retention and incontinency, constipation, tenesmus, diarrhea, venous obstruction, lymphedema, thrombophlebitis, sciatica and sciatic hernia (5). 

Distinguishing retroperitoneal lipomas from well-differentiated liposarcomas could prove difficult on imaging, and also biopsies may be confusing. Abdominal masses are frequently assessed by ultrasonography as the initial imaging modality. On ultrasound, lipoma appearance is of an oval hyperechoic mass with well-defined regular margins. Thin fibrous septa may be detected within the mass, and there is a lack of intra or perilesional vascularization on color Doppler examination (2). In present case, heterogeneous mass with lobulated border was found on ultrasound. Ultrasound findings per se, are insufficient for making a definitive diagnosis and there is a need for further imaging in the form of computed tomography (CT) or MRI scanning. CT scan can be used to diagnose a fatty tumor, but MRI, given its better soft tissue contrast resolution, is superior for specify of tissue. Another advantage of MRI over CT scan, is the former’s multiplanar imaging capability, which allows for detailed delineation of the anatomical extent of the mass, a crucial step for surgical preparations. On MRI, lipomas have specific appearance with their signal intensity being isointense with fat on all pulse sequences, with or without few thin linear internal septations (9). In this case, MRI showed a fat-containing dumbbell shaped lesion in the right pelvic side, suggestive for lipoma. On CT, lipomas appear as well-defined homogeneous masses with fat attenuation. Within the tumor, areas of soft-tissue attenuation indicating fat necrosis, septa, or normal adjacent structures might be observed. However, suspicious of liposarcoma should be raised in the presence of a mainly solid component or invasion of adjacent organ. It should be noted that any purely fatty lesion in retroperitoneum should be considered a well differentiated liposarcoma rather than a lipoma until histopathological examination proves it otherwise (10). CT findings of rapid growth, thick internal septa (>2mm), and solid components are indicative of malignancy (11). 

Percutaneous biopsy is controversial due to the risk of local spreading through the implantation of neoplastic cells along the route of the puncture, should it be a case of liposarcoma. Management of retroperitoneal lipomas is surgical resection and extreme caution to preserve the integrity of the capsule. Encapsulated presentation of the tumor presents a clear cleavage plane with surrounding structures, facilitating the surgical resection (12).

In gross examination, lipomas are encapsulated tumors with multi-lobed appearance resulting from yellowish rosy colored tumor with interspersed whitish fibrous septa, similar to our patient. It is noteworthy that low grade liposarcomas (grade I) may have the same delimited appearance (1).Less than 5% of all lipomas recur at a later stage, mostly as a result of incomplete excision (13), thus regular and prolonged surveillance should be considered. Repeated resections are considered a risk factor for malignant transformation of the lipoma (4). Ultrasonography, being repeatable and relatively inexpensive, is ideal for the follow-up of these patients (2). The present case was of a 66-year-old woman with abdominal pain and pelvic mass. Our literature review is based on search of relevant research publications found on SCOPUS, PubMed, UpToDate, Ovid and Google scholar databases from 1980 – 2020, using “retroperitoneal lipoma”, “pelvic mass” as keywords to search. To ensure that all potentially relevant researches are included, the reference lists of all retrieved articles were reviewed as well. In the review of medical literature, 14 cases of female patients with retroperitoneal pelvic lipoma were reported (table 1). The average age of patients was 44 years (13-76). In 7 cases, the patients were post-menopausal and the other 7 patients were less than 40 years old. Pain was the most common symptom in patients, while three of the 14 cases were asymptomatic and were discovered incidentally. In all cases, patients underwent resection of the tumor. 

In conclusion, in the differential diagnosis of retroperitoneal pelvic mass at all ages, lipoma should be considered as a rare cause.

**Table 1:** Fourteen retroperitoneal lipoma in female patients reported in the literature

**Treatment**	**Location**	**Symptom**	**Tumor size**	**Age**	**Author**
Laparotomy and tumor resection	Retroperitoneal mass in the right side of the pelvis	Abdominal pain, frequency and urgency	14×11×2 cm	66	Arab M et al., 2021
Laparotomy and tumor resection	Retroperitoneal fatty tissues, left ovary up to diaphragm	Abdominal distension and severe back pain, weight loss and constipation	45 × 48 × 13 cm	35	Mohammad Hasan M et al., 2019(14)
Laparotomy and tumor resection	Mesenteric tumor with adjoining bowel	Constipation, nausea, vomiting, abdominal and back pain	19.5 × 16.6 × 8.8 cm	13	Hardy et al., 2015(15)
Laparotomy and tumor resection	Right retroperitoneal fatty tissue	Slowly progressive abdominal pain and swelling	55× 40× 10 cm	70	Weniger et al., 2015(5)
Laparotomy and tumor resection	Intra-and extra-pelvic retroperitoneal space	Radicular sciatic pain followed by MRI revealed disc herniation and pelvic lipoma	6 × 13 × 15 cm	39	Duran et al., 2015(16)
Cesarean section with exploratory laparotomy and tumor resection	2.5 cm inferior to the lower pole of the left kidney	Massive painless mass	20× 12 × 10 cm	25	Wei D. *et al*., 2013(12)
Abdominal hysterectomy and tumor resection	Retroperitoneal space, from the floor and left lateral wall of pelvis	Urinary retention for 13 days	13.6 x 11.2 x 9.1 cm	36	Chander et al., 2012(3)
Exploratory laparotomy, tumor resection	Between the urinary bladder and the right iliac vessels in the right retroperitoneal pelvic space	Gluteal pain	15 cm	61	Ukita S. et al., 2009(17)
Exploratory laparotomy, tumor resection	Retroperitoneal space that pushed cecum towards anteromedial	Increasing swelling of limb, constipation	43x30x14 cm	67	Dirican A. et al., 2008(18)
Exploratory laparotomy, tumor resection	Left retroperitoneal space	Abdominal distention and early satiety	30 × 20 × 25 cm	50	Kansakar et al., 2007(19)
Exploratory laparotomy, tumor resection	Right iliac fosse	Abdominal pain and sickness	12 x 9 x 4 cm	72	Drop A. *et al*., 2003(20)
Exploratory laparotomy, tumor resection	Right iliac fossa and right flank, adherent to the right iliopsoas muscle and femoral nerve	Painless mass	20 x 13 x 10 cm	23	Martinez C. *et al*., 2003(1)
Laparotomy and tumor resection, high inguinal incision, A long femoral incision was necessary	Retroperitoneal mass extended down to the inguinal ligament, postero- lateral to the common femoral vessels	20-year history of a swollen right leg	20 x 20 x 12 cm	76	Acheson A. *et al*., 1997(21)
Exploratory laparotomy, tumor resection	Retroperitoneal mass in the pelvis with medial deviation of the distal right ureter	Intermenstrual bleeding	11 x 8 x 3 cm	26	Deppe G. *et al*., 1985(22)
